# Exploring the Effect of Milk Fat on Fermented Milk Flavor Based on Gas Chromatography–Ion Mobility Spectrometry (GC-IMS) and Multivariate Statistical Analysis

**DOI:** 10.3390/molecules29051099

**Published:** 2024-02-29

**Authors:** Chunlei Tan, Yang Tian, Liang Tao, Jing Xie, Mingming Wang, Feng Zhang, Zhijin Yu, Jun Sheng, Cunchao Zhao

**Affiliations:** 1 College of Food Science and Technology, Yunnan Agricultural University, Kunming 650201, China; tcl98316@163.com (C.T.); tianyang1208@163.com (Y.T.); taowuliang@163.com (L.T.); jingxie0624@163.com (J.X.); wmm295813@163.com (M.W.); 15762196909@163.com (F.Z.); yzj070517@163.com (Z.Y.); shengjunpuer@163.com (J.S.); 2 Engineering Research Center of Development and Utilization of Food and Drug Homologous Resources, Ministry of Education, Yunnan Agricultural University, Kunming 650201, China; 3 Key Laboratory of Precision Nutrition and Personalized Food Manufacturing, Ministry of Education, Yunnan Agricultural University, Kunming 650201, China; 4 PuEr University, PuEr 665000, China; 5 Yunnan Plateau Characteristic Agricultural Industry Research Institute, Kunming 650201, China

**Keywords:** milk fat, fermented milk, response surface methodology, GC-IMS, volatile compounds, multivariate statistical analysis

## Abstract

Milk fat is a premium nutritional health product, yet there is a lack of high-fat dairy products for daily consumption in the current market. This study investigated the influence of different milk fat contents on the physicochemical and textural properties of fermented milk. The research revealed that an increase in milkfat content significantly improved the water-holding capacity, syneresis, color, hardness, springiness, gumminess, and chewiness of fermented milk, while showing minimal changes in pH and total titratable acidity. Response surface analysis indicated that fermented milk with 25% milk fat, 2.5% inoculum, a fermentation time of 16 h, and a fermentation temperature of 30 °C exhibited the highest overall acceptability. Using GC-IMS technology, 36 volatile compounds were identified, with an increase in milk fat content leading to elevated levels of ketone compounds, and 14 compounds were defined as key aroma compounds (ROAV > 1). Electronic nose distinguished samples with different milk fat contents. The results demonstrate that an increase in milk fat content enhances the physicochemical and flavor attributes of fermented milk. This work provides theoretical references for the production and development of high-fat fermented milk.

## 1. Introduction

Apart from being crucial for the physicochemical, structural, and flavor characteristics of dairy products, milk fats are also an excellent source of high-quality nutrients that are beneficial for human consumption, especially for the growth and development of children. [1,2,3]. The presence of palmitic acid at the sn-2 position in milk fat has been found to promote nutrient absorption in infants, enhance bone growth, and contribute to brain development [4]. Additionally, milk fat is the richest source of conjugated linoleic acid (CLA), which is believed to possess anti-mutagenic, anti-tumor, and anti-atherosclerotic properties. Fermenting milk into yogurt or cheese has been shown to significantly increase the content of CLA [5]. The breakdown and oxidation of milk fat can impact the flavor profile of fermented milk, with the formation of flavor compounds such as ethyl acetate and 2-heptanone closely associated with milk fat [6]. Furthermore, the degradation of milk fat generates fatty acids, particularly short-chain fatty acids, which contribute to the unique flavor of matured Kope cheese [7]. Moreover, milk fat can increase the hardness and adhesiveness of yogurt, reduce whey separation by enhancing water-holding capacity, and improve product stability [8]. However, high-fat dairy products currently available on the market, such as cream, cheese, and butter, are more common in European countries than in China, where traditional dietary habits do not frequently include products made from milk fat, leading to a gap in the domestic market for milk fat-based dairy products.

Fermented milk, a nutritionally rich dairy product produced by fermenting milk with lactic acid bacteria, is widely consumed, and its demand continues to grow globally due to its tangy flavor and aromatic qualities [9]. Traditional fermented milk is usually made with fresh milk as a raw material, adding sucrose through a series of processes such as preheating, homogenization, sterilization, inoculation, subpackaging, fermentation, and refrigeration [10]. Depending on the technology, it can be divided into solidified and stirred fermented milk [11]. However, the current market lacks fermented milk products with high milk fat content, which could enhance flavor and potentially influence consumer preferences. Research suggests that the health impact of dairy products is more dependent on type rather than fat content, with evidence indicating that the consumption of fermented milk and cheese, which are rich in milk fat, may have preventive effects against heart disease [12]. Therefore, the development of fermented milk products with high milk fat content is of significant importance.

Advancements in omics technologies have led to the widespread application of sensory omics analysis in the study of dairy product flavors. Studies using GC-MS have revealed differences in volatile compounds between full-fat yogurt and other yogurt types [13], and electronic tongue analysis has shown correlations between sourness and sweetness in yogurt [14]. Furthermore, PTR-TOF-MS research has identified changes in flavor compounds with increasing milk fat content [15]. However, traditional odor analysis methods have limitations in the extraction, separation, and quantification of volatile compounds in fermented milk. Gas chromatography-ion mobility spectrometry (GC-IMS) has emerged as a new technology for accurate quantitative and qualitative analysis of volatile flavor compounds in food [16], yet its application in exploring the influence of milk fat on the volatile compounds in dairy products is limited [17].

This study evaluates the influence of milk fat content on the physicochemical and textural properties of fermented milk, optimizing the fermentation process and investigating the impact of various factors (starter culture inoculation, milk fat content, fermentation temperature, and time) on sensory evaluation. Additionally, the study explores the changes in volatile components within different milk fat content ranges. The findings of this research will provide a new theoretical basis for the development of high-quality fermented milk products and enhance our understanding of the relationship between milk fat content and flavor in fermented milk.

## 2. Results and Discussion

### 2.1. Effects of Milk Fat on pH and Total Titratable Acidity of Fermented Milk

**Figure 1:**
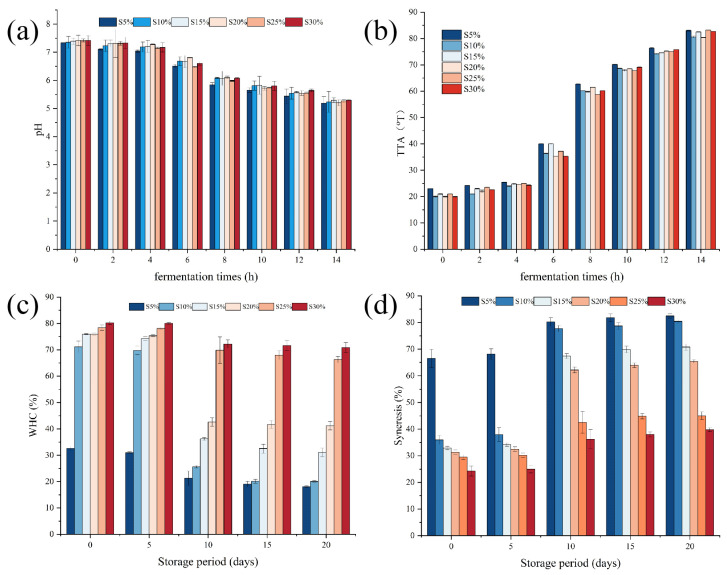
pH (**a**) and total titratable acidity (**b**) of fermented milk with different milk fat additions during fermentation and water holding-capacity (WHC) (**c**) and syneresis (**d**) during storage.

Figure 1a,b illustrate the variations in pH and total titratable acidity that occur throughout the fermentation process. The pH and TTA values of the six groups of fermented milk samples exhibited comparable trends during the fermentation process as the fat content increased. According to He et al., this is because lactic acid, which is created by lactic acid bacteria during anaerobic respiration throughout the production process, is the primary source of the pH drop during yogurt fermentation [18]. Since the fermentation of fermented milk primarily uses carbohydrates and is unrelated to fat, the pH values of the various samples were essentially the same, and the pH values decreased as the fermentation process progressed. Consistent with the trend of pH changes, the TTA of all samples gradually increased as fermentation progressed. In the early stage of fermentation, the increase in TTA was not significant within 0 to 4 h but became obvious within 4 to 8 h. This may be due to the significant increase in the anaerobic respiration of lactic acid bacteria and the conversion of a large amount of lactose into lactic acid [18], resulting in a significant decrease in the pH of fermented milk and a rapid increase in TTA.

### 2.2. Effects of Milk Fat on Water-Holding Capacity, Syneresis, and Color of Fermented Milk

Water-holding capacity is one of the important indicators in evaluating the stability of fermented milk [19]. As shown in Figure 1c, with the increase of fat content, the WHC of the fermented milk showed an increasing trend. When the fat content increased from 5% to 10%, the WHC of the fermented milk significantly increased from 32.62% to 71.11%, and reached a peak of 82.24% when the fat content was 30%. In the first 5 days, there was little change in the WHC of all samples. From day 5 to 10, the WHC of the samples with 5%, 10%, 15%, and 20% fat content significantly decreased, with the WHC of the 10% fat content sample dropping from 69.75% to 25.58%. The WHC of the fermented milk remained relatively stable from 10 to day 20. When the fat content reached 25% and 30%, the WHC of fermented milk samples showed minimal changes during storage. This is attributed to the increased fat content, which enhances the ability of the fermented milk to bind water molecules, making its gel system more stable [20].

Syneresis reflects the compactness of the structure of the fermented milk [21]. Higher syneresis indicates that the water in the fermented milk is easily lost, resulting in a loose structure. Lower syneresis indicates a tighter structure and better stability. As shown in Figure 1d, corresponding to the water-holding capacity, the syneresis of the fermented milk gradually decreased with the increase in fat content. From day 5 to 10, the syneresis of the samples with 5%, 10%, 15%, and 20% fat content significantly increased. Throughout the storage period, the syneresis of the 25% and 30% fat content samples showed minimal changes. This is because with the increase in fat content, the interaction between fat and protein makes the structure of the fermented milk more compact, and the water is less likely to be lost. The results indicate that S25% and S30% exhibit the best water-holding capacity and syneresis.

In addition to influencing consumer acceptability and choice, color serves as a crucial point of reference for the development of new dairy products. White, for instance, can assist in conveying freshness and purity in dairy products like yogurt and hence meet customer purchasing criteria. As shown in Table S1, with the increase in fat content, the L* value of the fermented milk gradually increased, and during storage, the L* value of the fermented milk samples showed an upward trend, while the a* value showed a downward trend. The a* values of S5% and S10% continuously decreased during storage, while the other four groups first increased and then decreased. When the fat content was between 5% and 15%, the b* value of the fermented milk continued to decrease. When the fat content reached 25%, the b* value increased relative to 4%, and when it reached 30%, the b* value reached its maximum. During storage, the b* and a* increased first, then decreased, and the L* showed a consistently increasing trend.

### 2.3. Effects of Milk Fat on Texture of Fermented Milk

The cohesiveness, gumminess, chewiness, hardness, and springiness of fermented milk can all be indicators of its structure and texture [22]. To obtain the required thickening effect in fermented milk, manufacturers frequently add emulsifiers or thickeners such as pectin, arabic gum, and gelatin. Nevertheless, the texture of the fermented milk may suffer if thickeners or emulsifiers are added in excess. As a result, it makes sense to change the material content of milk. Table 1 illustrates how the hardness, gumminess, springiness, and chewiness of fermented milk rose as the fat level increased, while the cohesiveness was barely altered. This is because an increase in fat content has little effect on the cohesiveness of fermented milk, which is mostly due to the inherent viscosity of poly-saccharides and their interaction with the protein matrix [19]. The chewiness and hardness of fermented milk are directly correlated to its texture. Fermented milk will appear mushy and have trouble forming a coagulum if the hardness is too low. Poor texture results from the fermented milk losing its distinct viscosity due to excessive hardness. Therefore, the hardness of the fermented milk was most appropriate at 15%, 20%, and 25% fat contents. The gumminess and springiness of fermented milk affect its chewiness. As the fat content increased, the gumminess of fermented milk increased with the increase in the density of raw milk. After homogenization and other mechanical treatments, the fat is broken into small fat globules, which interact with proteins during lactic acid fermentation, increasing the hardness and cohesiveness of the fermented milk and, thus, the chewiness [22]. Therefore, when the fat content reached 15%, 20%, and 25%, the tissue structure of the fermented milk improved, and the texture was more satisfactory.

### 2.4. Results of the Single-Factor Experiment and Response Surface Methodology

**Figure 2:**
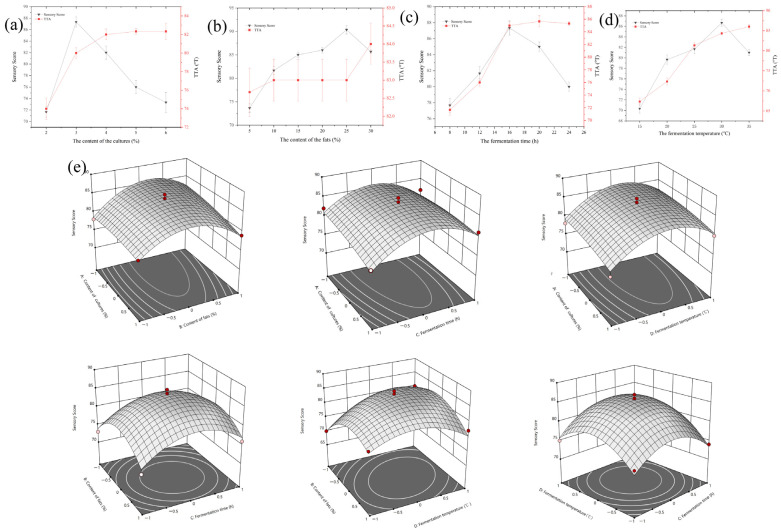
The effect of culture content (**a**), milk fat content (**b**), time (**c**), and temperature (**d**) on the fermented milk samples and response surface plots (**e**) of the effects of variables on the sensory score of the fermented milk.

The total titratable acidity content has a significant impact on the flavor and quality of fermented milk products [23], with sensory indicators being the main factors influencing consumer choices of yogurt and fermented milk products [24]. The results of the single-factor experiments are shown in Figure 2. As the amount of starter culture inoculation increased, the sensory scores of the fermented milk showed a trend of initially increasing, then decreasing, with acidity increasing with the amount of starter culture inoculation. When the inoculation amount was less than 3%, the TTA of the fermented milk was lower, indicating incomplete fermentation (Figure 2a). However, when the inoculation amount exceeded 3%, further increases did not significantly affect the acidity of the fermented milk, and the sensory scores were highest at 3% inoculation. Thus, an inoculation amount of 3% may be considered appropriate. As shown in Figure 2b, with an increase in milk fat content, the TTA of the fermented milk did not significantly change. This phenomenon is explained in Section 3.1. Additionally, an appropriate increase in milk fat content led to significant improvements in the appearance, texture, and taste of the fermented milk. When the milk fat content reached 25%, the overall sensory score of the fermented milk was optimal. The 25% milk fat content exhibited noticeable gumminess during stirring, a rich milky aroma, suitable springiness, and a pleasant sweet–sour taste. This is due to the production of various precursor compounds of flavor components through the hydrolysis of milk fat and proteins during fermentation, as well as the generation of various flavor compounds through probiotic metabolism. Higher fermentation temperatures result in increased TTA in the fermented milk. An appropriate fermentation temperature is conducive to the growth of probiotics and accelerates the acid production rate. When the temperature reached 30 °C, the sensory scores of the resulting fermented milk were optimal (Figure 2c). As shown in Figure 2d, the TTA of the fermented milk increased with prolonged fermentation time. Between 12 and 16 h, the TTA increased rapidly as the lactic acid bacteria grow rapidly. After 16 h, the rate of acidity increase became gradual, and the sensory scores of the fermented milk reached their peak at 16 h.

Table S2 shows the results of the RSM, the quadratic equation model for the sensory score of fermented milk in relation to the inoculation amount (A), milk fat content (B), fermentation temperature (C), and fermentation time (D) obtained through multivariate regression fitting of Table 2 according to Equation (1):

Sensory Score = 85.60 − 0.92A + 1.00B + 0.58C + 1.67D − 1.00AB + 0.75A + 0.25BC − 2.75BD + 0.75CD − 0.67A^2^ − 5.3B^2^ − 5.18C^2^ − 5.05D^2^. (1)

The regression results indicated that the model’s differences were statistically significant (*p* < 0.0001), with an F value of 19.71. The predicted values aligned well with the actual values (R^2^ = 0.95). The adjusted r^2^ was 0.9034, indicating that only 9.66% of the variance cannot be predicted by this model. The insignificant fitting discrepancies further demonstrated the model’s good accuracy.

Combining Table 2 and the regression model, it is evident that the single-factor impact on the sensory score of high-fat fermented milk occurs in the following order: fermentation temperature (D) > milk fat content (B) > starter culture inoculation amount (A) > fermentation time (C). In order to better explain the complex relationships between different variables, the response surface plot shown in Figure 2e provides a clearer understanding. Based on the actual situation, the optimal addition formula and fermentation process were determined to be 25% milk fat, 2.5% starter culture, and a fermentation time of 16 h at a fermentation temperature of 30 °C. Therefore, the appropriate fermentation conditions mentioned above are conducive to producing high-fat fermented milk products with optimal sensory qualities. When the milk fat content was 25%, the physicochemical properties and sensory scores of fermented milks reached optimal values; thus, subsequent experiments analyze S25% and the control group (S5%).

### 2.5. Analysis of E-Nose

**Figure 3:**
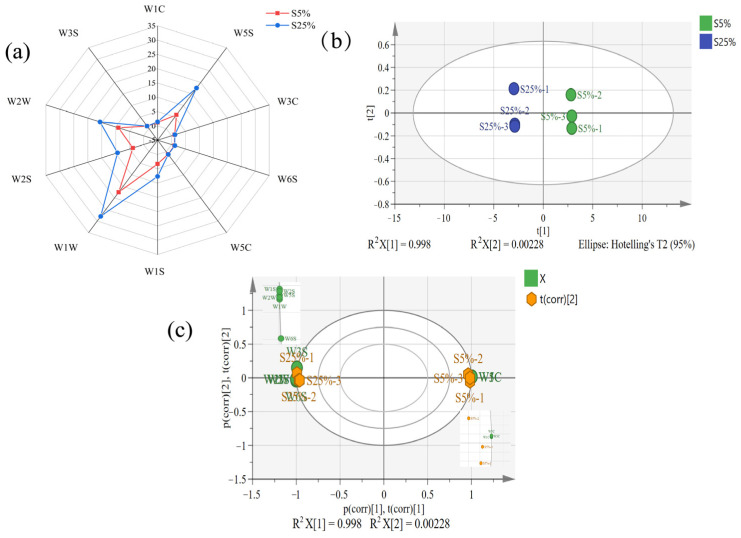
Radar image (**a**), biplot (**b**), and loading plot (**c**) of principal component analysis using an E-nose on fermented milk. S5% and S25% represent fermented milk samples with 5% and 25% milk fat content, respectively. The types of sensors corresponding to each ID could be found in Table S5.

Electronic noses simulate human olfaction and are widely used in dairy product testing [25]. Throughout the entire testing process, the response values of sensors W5S, W1S, W1W, W2S, and W2W were significantly higher than those of other sensors, with W1W showing the strongest response (Figure 3a). After the increase in fat content, the sensor response types of both sample groups were the same, but the response values were significantly different. Compared to the S5% samples, the response values of sensors W5S, W2W, and W1W to the S25% samples were much higher. This indicates that the fat content affects the formation of short-chain hydrocarbons in fermented milk, such as nitrogen oxides, methane, sulfur gases, alcohols, ethers, ketones, and aldehydes. To further explore the differences between the fermented milk samples, a PCA model was established to differentiate the samples. From Figure 3b, it can be seen that the PCA model could cover most of the original information of the samples, and the two groups could be significantly separated. S5% was located in the positive half of the horizontal axis, while S25% was located in the negative half, and the two samples were significantly distinguished. This indicated that an E-nose can effectively differentiate between the two types of fermented milk, and the odor of S25% fermented milk was significantly higher than that of S5% fermented milk. From Figure 3c, it can be observed that the electronic nose sensors can be clustered into two groups. W1C, W5C, and W3C form one cluster located on the positive half of the PC1 axis, positively correlated with S5%. W1S, W2W, W2S, W5S, W1W, and W6S form another cluster located on the negative half of the PC1 axis, positively correlated with S25%. This indicates that an increase in milk fat content may lead to an enhancement of aromatic components in fermented milk, thereby improving the aroma of fermented milk.

### 2.6. Analysis of GC-IMS

#### 2.6.1. The Volatile Components in Two Fermented Milks Identified by GC-IMS

**Figure 4:**
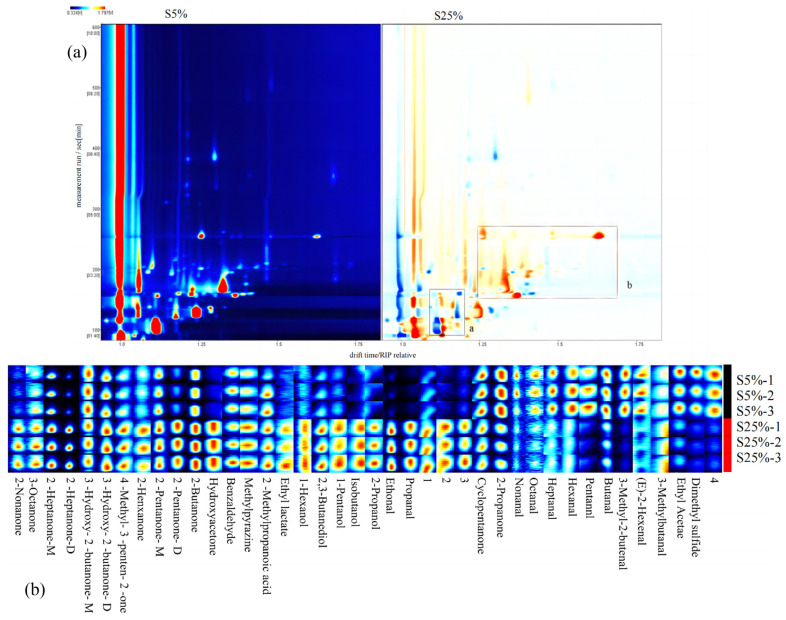
(**a**) Differentiation plot of volatile compounds. In S25%, red and blue dots indicate that the concentration of the compounds is higher and lower than S5%, respectively. (**b**) Gallery plot fingerprint of different fermented milks by GC-IMS. S5% and S25% represent fermented milk samples with 5% and 25% milk fat content, respectively.

Aroma is an important factor in determining the quality of fermented milk, and the aroma components of fermented milk are a mixture of various components [26]. In this study, a total of 36 volatile compounds were accurately detected in fermented milk using GC-IMS (Table 3), including 14 ketones, 11 aldehydes, 6 alcohols, and 5 other components. The red vertical line at coordinate 1.0 in Figure 4a represents the RIP peak (reaction ion peak), with each point on either side of the peak representing a volatile compound, where darker colors and larger areas of the points indicate higher contents [27]. It can be seen from Figure 4a that in region a, the component content in S25% is lower than that in S5%. In region b, the component concentration in S25% is significantly higher than that in the S5% samples. The ketones were found to be the most abundant volatile flavor components (Table 3). Among them, ketones in S5% accounted for 90.16% of the total component content, while ketones in S25% accounted for 81.77%. Aldehydes, alcohols, and other components in S5% accounted for 5.12%, 3.06%, and 1.66% of the total component content, respectively. In S25%, aldehydes, alcohols, and other components accounted for 5.97%, 11.2%, and 1.06% of the total component content, respectively. Ketones in fermented milk mainly come from the β-oxidation of fatty acids [28], and the aroma of ketones becomes more intense as the carbon chain lengthens and the branched chain increases. According to previous studies, 3-hydroxy-2-butanone and 2-heptanone are sources of a creamy aroma in fermented milk. In addition, 2-butanone is a source of fruit flavor, 2-pentanone is a source of sweetness, and 2-nonanone is a source of cheese flavor [29]. Due to the increase in milk fat content in the S25% sample, the substrate for ketone synthesis increased, resulting in a significantly higher ketone content in S25% compared to S5%. According to the research of Liu et al. [30], the formation of aldehydes in yogurt is mainly caused by the oxidation of unsaturated fatty acids. Aldehydes have a low flavor threshold and distinct flavor characteristics, playing an important role in yogurt flavor. For example, nonanal is the main source of floral, citrus, and fatty aromas in yogurt, while benzaldehyde is the source of bitter almond and burnt flavors. The formation pathway of alcohols is mainly the reduction reaction of aldehydes. Ethanol has a slightly sweet and spicy taste, and 1-pentanol has a spicy and wine-like taste [31]. The presence of alcohols makes the flavor of fermented milk more intense. In addition, the formation pathway of esters is mainly the esterification reaction between milk fatty acids and alcohols, which can give fermented milk a fruity aroma.

The use of fingerprinting and clustering heat maps can more intuitively distinguish the differences between samples. Figure 4b shows that the S5% sample has higher levels of cyclopentanone, acetone, nonanal, octanal, heptanal, hexanal, pentanal, butanal, 3-methyl-2-butenal, E-2-hexenal, 3-methylbutanal, ethyl acetate, and dimethyl sulfide. In S25%, the levels of methylpyrazine, 2-methylpropanoic acid, ethyl lactate, 1-hexanol, 2,3-butanediol, 1-pentanol, isobutanol, 2-propanol, ethanol, and propanal were higher. The content of ketone compounds in S25% was much higher than that in S5%, mainly due to the higher milk fat content in S25%, which provides more free fatty acids for β-oxidation reactions and generates more ketone compounds. Figure S1 shows that the contents of 2,3-butanediol, 2-butanone, 3-hydroxy-2-butanone-D, hydroxyacetone, ethanol, propanal, 2-nonanone, 2-octanone-M, 2-octanone-D, 2-pentanone-M, 2-pentanone-D, and 4-methyl-3-penten-2-one in S25% were higher than those in S5%. The relative content of aldehydes such as pentanal, hexanal, heptanal, and butanal was higher in the S5% sample. This is consistent with the results of fingerprinting, indicating that adding a certain amount of milk fat can increase the concentration of ketones in fermented milk, possibly making it sweeter and fruitier.

#### 2.6.2. Multivariate Statistical Analysis by GC-IMS

**Figure 5:**
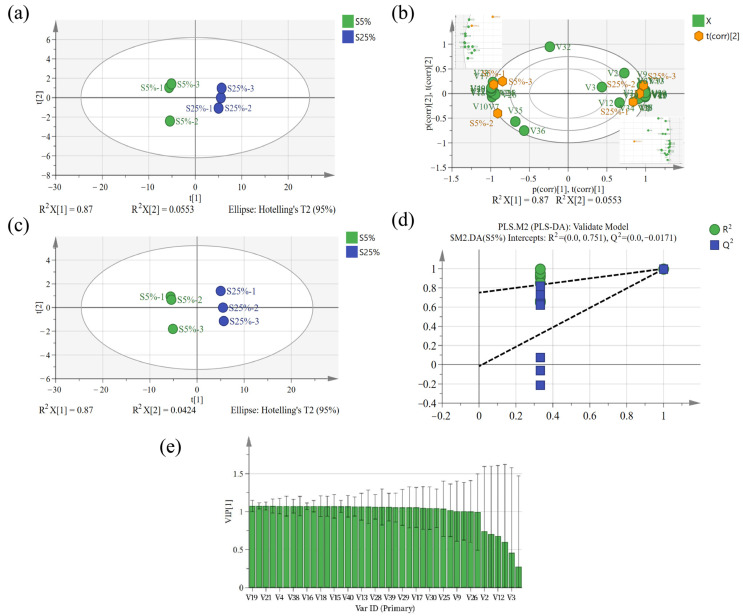
Multivariate analysis of aroma compounds in fermented milks based on GC–IMS: the 2D scores (**a**) and the loading plots (**b**) analyzed by PCA, as well as the 2D scores (**c**), a permutation plot tested 1000 times (**d**), and a graph of the VIP score (**e**) for volatile compounds by PLS-DA. S5% and S25% represent fermented milk samples with 5% and 25% milk fat content, respectively. The volatile flavor compounds corresponding to each ID could be found in Table 3.

To further explore the influence of different milk fat contents on the flavor of fermented milk, PCA and PLS-DA models were established using SMICA 14.1 software, and the peak intensity values of the characteristic peaks of two fermented milk samples were selected as the feature parameter variables for analysis. It is generally believed that the PCA model has good explanatory power when the cumulative contribution rate reaches 60% [32]. As shown in Figure 5a, the contribution rates of PC1 and PC2 are 87% and 5.53%, respectively, and the sum of the contribution rates of PC1 and PC2 is 92.53%, indicating that the two principal components can sufficiently reflect the information reflected by the original data. S5% and S25% were well separated on the two principal components, indicating the presence of flavor differences between the two sample groups. In Figure 5b, the score plot of the PCA model can better analyze the correlation between compounds and samples. 1-pentanol, 4-methyl-3-penten-2-one, isobutanol, ethanol, 1-hexanol, ethyl lactate, 2,3-butanediol, 2-butanone, acetaldehyde, hydroxyacetone,3-hydroxy-2-butanone-D, 2-nonanone, 2-heptanone-D, 2-pentanone-D, 2-heptanone-M, and 2-propanol were strongly correlated with the S25% sample on the positive half of the abscissa axis; ethyl acetate, butyraldehyde, dimethyl sulfide, 2-propanone, 3-methyl-2-butenal, 3-hydroxy-2-butanone-M, pentanal, cyclopentanone, hexanal, heptanal, nonanal, and octanal were positively correlated with the S5% sample, and these compounds can be used as identifying compounds for the S5% sample.

A supervised PLS-DA model was further established to differentiate the volatile compounds in the two groups of fermented milk samples. Consistent with the results of the PCA analysis, S5% and S25% were also significantly distinguished (Figure 5c). In this PLS-DA analysis, the fit index of the independent variables (R^2^x) was 0.87, the fit index of the dependent variables (R^2^y) was 0.997, and the model prediction index (Q^2^) was 0.995, demonstrating the excellence of the model. After 200 permutation tests, as shown in Figure 5e, the intercept of the regression line of Q^2^ was less than zero, and the original Q^2^ value was greater than all the Y vectors, proving the reliability of the model. The variable importance in projection (VIP) of the PLS-DA model can indicate the degree of difference in the impact of volatile substances on sample flavor. Compounds with VIP > 1.05 are generally considered to be differential compounds. As shown in Figure 5d, 24 types of substances including 2-nonanone (VIP = 1.078), 2-heptanone-M (VIP = 1.079), 2-heptanone-D (VIP = 1.078), 2,3-butanediol (VIP = 1.076), 3-hydroxy-2-butanone-M (VIP = 1.050), 3-hydroxy-2-butanone-D (VIP = 1.077), 4-methyl-3-penten-2-one (VIP = 1.071), 2-pentanone-D (VIP = 1.076), 2-pentanone-M (VIP = 1.068), 2-butanone (VIP = 1.077),butanal (VIP = 1.061), 2-propanone (VIP = 1.076), propanal (VIP = 1.080), hydroxyacetone (VIP = 1.079), 3-methyl-2-butenal (VIP = 1.079), ethyl acetate (VIP = 1.054), pentanal (VIP = 1.061), (E)-2-hexenal (VIP = 1.071), dimethyl sulfide (VIP = 1.069), 2-propanol (VIP = 1.062), 1-pentanol (VIP = 1.050), isobutanol (VIP = 1.076), and 1-hexanol (VIP = 1.069) were selected as differential compounds, and the differences in their contents potentially characterize the influence of milk fat on the flavor of fermented milk.

#### 2.6.3. The Key VOC Analysis by ROAV

The contents of aroma compounds cannot be used as the sole basis for their overall aroma contribution, as it is also influenced by their sensory thresholds. The ROAV value, which is a comprehensive measure of the concentration of a substance and its threshold, is an effective method for identifying key aroma compounds [33]. For example, 3-hydroxy-2-butanone-D, with a relatively high relative content and low threshold in fermented milk samples, was assigned an ROAV value of 100. As shown in Table S3, two groups of fermented milk samples had a total of 14 volatile compounds with ROAV values greater than 1, including eight ketones and six aldehydes. Among them, compounds such as 3-hydroxy-2-butanone-M (42.134~64.152), 3-hydroxy-2-butanone-D (100), and butanal (41.691~87.690) exhibited high ROAV values, suggesting that these components may make significant aroma contributions to fermented milk. From Table S5, it can be observed that the types of compounds with ROAV values greater than 1 changed between S25% and S5% samples, and the ROAV values of individual components also changed. With an increase in milk fat content, the ROAV values of 2-octanone-D and propanal, which are unique key aroma components in S25%, increased from 0.77 and 0.313 to 2.250 and 1.786, respectively. Conversely, the ROAV values of 2-propanone and octanal decreased from 1.71 and 1.485 to 0.77 and 0.586, respectively, in the S5% sample. Additionally, the ROAV values of 2-octanone-M, 2-pentanone-D, and 2-hexanone increased, while those of benzaldehyde, 3-hydroxy-2-butanone-M, 2-pentanone-M, butanal, 3-methylbutanal, and nonanal decreased, reflecting changes in their relative contents in the fermented milk. Ketones and aldehydes, due to their unique flavors and low thresholds, are important sources of fermented milk flavor composition [34]. In this study, 3-methylbutanal (ROAV: 1.368~2.104) was found to have a fruity aroma and is a primary product of the Maillard reaction, while nonanal (ROAV: 2.918~4.752) is a key aroma component in cheese, imparting a fatty aroma to dairy products [35]. Overall, changes in milk fat content altered the flavor characteristics of fermented milk to varying degrees, consistent with the results of electronic nose analysis.

### 2.7. Correlation Analysis of VOCs with an E-Nose

**Figure 6:**
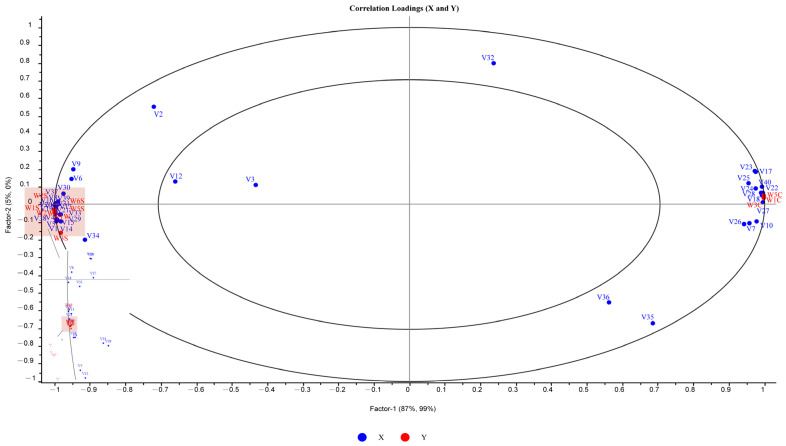
Correlation loading plot for volatile compounds (X matrix) and E-nose of fermented milks (Y matrix). Blue and red represent the VOCs and E-nose, respectively. The volatile flavor compounds and the types of sensors corresponding to each ID could be found in Table 3 and Table S5, respectively.

A partial least squares regression (PLSR) model was established to analyze the correlation between the peak areas of detected volatile compounds in fermented milk and the response values of an electronic nose sensor for different milk fat contents. The PLSR model revealed that the two principal components of the x variable (volatile compounds) explained 93% of the variance, while the y variable (E-nose sensor) explained 99% of the variance; the size ovals represent variances of 50% and 100%, respectively [36]; and all response values fall within the two circles. The distribution of the sensors and their correlation with specific volatile compounds are visualized in Figure 6. Sensors W1C, W3C, and W5C are located on the right side of the plot, while the other seven sensors are on the left side. A close proximity of a sensor to a volatile compound in the plot indicates a higher correlation. Sensors W1C, W5C, and W3C were found to characterize the S5% sample, primarily discriminating between the two groups of samples based on the PCA analysis. Sensor W1C showed a good correlation with V18 (2-propanone), sensor W5C with V28 (dimethyl sulfide) and V22 (3-methyl-2-butenal), and sensor W3C with V27 ((*E*)-2-hexenal). Additionally, sensor W6S showed a good correlation with V20 (propanal) and V11 (3-hydroxy-2-butanone-D), and sensor W1W with V4 (2-heptanone-M). These results indicate that ketones and aldehydes were the main compounds causing changes in the response values of the electronic nose sensor. This finding is consistent with the conclusion of GC-IMS that ketones and aldehydes are the two compounds with the highest contents and provide the key aroma in fermented milk. As indicated by the diagram, these two types of compounds are also key compounds that distinguish fermented milk with different milk fat contents.

## 3. Materials and Methods

### 3.1. Materials and Reagents

Fresh raw milk (Songming Ranch, Kunming, China); white granulated sugar (Gengma Nanhua Sugar Co., Ltd., Lincang, China); and starter cultures containing *Lactobacillus delbrueckii* subsp. *Bulgaricus*, *Streptococcus thermophilus*, and *Lactobacillus casei* Zhang (Kohansen Trading Co., Ltd., Beijing, China) were used in this study. C5~C25 n-alkanes (chromatographically pure) were purchased from Beijing Chemical Reagent Co., Ltd. (Beijing, China).

### 3.2. Fermented Milk Preparation

Figure S2 illustrates the fermented milk preparation method. To obtain milk fat, fresh cow’s milk was centrifuged at 40 °C for 20 min at 4000 rpm to degrease it. After that, skim milk was supplemented with milk fat to achieve 5%, 10%, 15%, 20%, 25%, and 30% milk fat contents. The remaining ingredients remained the same. The reset milk was heated to 45 °C, sugar (8.5 g/100 g) was added, and it was stirred for 10 min. After that, the temperature was raised to 65 °C to homogenize the milk, followed by pasteurization for 15 min at 85 °C and cooling to 40 °C for inoculation with the 2% (*w*/*w*) fermentation agent. To obtain fermented milk samples, the milk was first fermented at 30 °C for 14 h. It was then allowed to mature at 4 °C for 10 h. The six groups of samples were then designated as S5%, S10%, S15%, S20%, S25%, and S30% and stored at 4 °C.

### 3.3. Physicochemical Determinations

The following procedure was used to measure the pH value. After standing for 30 min, the pH meter electrode was placed in a conical flask containing 2.00 g (accurate to 0.01, mixed with 50 mL distilled water) of fermented milk and mixed uniformly. The pH value was recorded once the reading was steady. Potentiometric titration was used to measure the total titratable acidity (TTA) of fermented milk [23], computed according to Equation (2) as follows:

(2) To=(V1−V0)×CNaOH×100m×0.1

where (*V*1 − *V*0) is the volume of NaOH consumed when titrating the sample from the initial pH to 8.3, *CNaOH* denotes the molar concentration of standard alkali NaOH, and m denotes the mass of the test sample. The acidity of fermented milk is defined as the volume of 0.1 moL·L^−1^ NaOH consumed per 100 g of milk.

The L* (lightness), a* (redness/greenness), and b* (yellowness/blueness) of the fermented milks were measured by a colorimeter. The method of Ge et al. was used to determine the water-holding capacity (WHC) [20]. After adding 8 g of fermented milk to an empty 10 mL centrifuge tube (*W*1) and measuring total mass as *W*2, the tube was centrifuged for 10 min at 3000 r/min. The supernatant was then discarded after standing at room temperature for 15 min. Note the precise mass of the leftover material, denoted as *W*3. The *WHC* was determined according to Equation (3) as follows:

(3) $$WHC\;(\%)=\frac{W3-W1}{W2-W1}\times 100$$

Syneresis was determined using the method proposed by Molaee et al. [21]. A centrifuge tube containing 20 g of fermented milk was centrifuged for 8 min at 3000 rpm. After allowing the centrifuge tube to stand at room temperature for 2 min, the weight of the supernatant was measured and recorded. The *syneresis* was determined according to Equation (4) as follows:

(4) $$Syneresis(\%)=\frac{m1}{m2}\times 100$$

where *m*1 is the mass of the fermented milk sample, and *m*2 is the mass of the fermented milk supernatant.

### 3.4. Texture Profile Analysis

To ascertain the texture of fermented milk, samples of fermented milk were held at room temperature for 20 min after being chilled for 24 h at 4 °C. An FTC texture analyzer in TPA compression mode with a P/5 cylindrical probe was used to measure the texture. The speed before the test was 120 mm/min, the speed during the test was 60 mm/min, and the speed after the test was 120 mm/min. First, we set the parameter to 50% of the compression deformation. The initial force was 0.02 N. Lastly, characteristics like chewiness, gumminess, cohesiveness, hardness, and springiness were measured, with a total of 5 measurements per sample [37].

### 3.5. Experimental Design of Single-Factor Experiments and Response Surface Methodology

The factors affecting the TTA and sensory score of fermented milk were evaluated using single-factor experiments. Milk fat concentration (5, 10, 15, 20, 25, and 30%), culture amount (2, 3, 4, 5, and 6%), fermentation temperature (15, 20, 25, 30, and 35 °C), and fermentation time (8, 12, 16, 20, and 24 h) were chosen as the assayed factors. Four factors and three levels of the response surface methodology (RSM) were designed to determine the optimal conditions of fermented milk production. The sensory properties of fermented milks were evaluated with reference to Ye et al. [38]. The RSM test parameters and levels are shown in Table S4.

### 3.6. Electronic Nose (E-Nose) Analysis

Ten distinct metal oxide sensors (W1C, W5S, W3C, W6S, W5C, W1S, W1W, W2S, W2W, and W3S) were tested using the PEN3 electronic nose system (AIRSENSE, Schwerin, Germany); the sensors are listed in Table S5. The assay was carried out by directly inserting a syringe needle into the headspace flask containing the sample after 10 g of fermented milk was placed in a 100 mL beaker and allowed to stand at room temperature for 30 min, in accordance with Yan et al.’s process [25]. The injection flow rate was 400 mL/min, the sample preparation time was 5 s, the sensor zeroing time was 5 s, the sampling time for analysis was 1 s/group, and the sensor self-cleaning time was 80 s.

### 3.7. Analysis of Volatile Compounds (VOCs) by GC-IMS

The VOCs of fermented milk samples were examined using a GC-IMS instrument (FlavourSpec^®^, Dortmund, Germany) fitted with an MXT-5 chromatographic column (15 m × 0.53 mm × 1 μm, RESTEK, Bellefonte, PA, USA). The analysis method is mostly based on and slightly modified from the techniques of Zhang et al. [39] and Leng et al. [40]. A total of 5 g of fermented milk was added to a 20 mL headspace bottle and incubated at 40 °C for 15 min. A syringe set at 85 °C was then used to automatically inhale the top gas, and 99.999% nitrogen was utilized as drift and carrier gas. The flow rate parameter was 2 mL/min for 2 min, then rose linearly to 150 mL/min in 18 min and to 150 mL/min in 20 min. The IMS temperature was 45 °C during the 20 min analysis period, and 60 °C was the temperature in the column. By contrasting the standard drift times in the retention index and GC-IMS libraries, VOCs were found. Flavor compounds were subjected to a qualitative study using the NIST and IMS databases. The relative content of VOCs was compared quantitatively using the peak area signal intensity that was produced using LAV software.

The contribution of volatile compounds to sample flavor can be calculated using *ROAV*. The volatile compounds with *ROAV* > 1 are often thought to be the main flavoring compounds in the sample, whereas the volatile compounds with 0.1 < *ROAV* ≤ 1 are thought to alter the sample’s overall flavor [41]. The *ROAV* was calculated by the following Equation (5):

(5) $$ROAVi=\frac{Ci}{Cmax}\times \frac{Tmax}{Ti}\times 100\%$$

where *Ci* and *Cmax* are the relative content of each component and the relative content of the compounds that contribute the most to the overall flavor, respectively; *Ti* represents the sensory threshold of each component; and *Tmax* is the sensory threshold of the compound that contributes the most to the overall flavor.

### 3.8. Statistical Analysis

Excel was used to organize the data, and SPSS Statistics 27 was used for ANOVA and significance analyses. *p* < 0.05 was regarded as statistically significant. The 2D and 3D spectra of VOCs were created using the Reporter plugin of LAV, the instrument’s accompanying software, and their fingerprints and difference maps were produced using the Gallery Plot plugin. The fermented milk samples were subjected to principal component analysis (PCA) and partial least squares discriminant analysis (PLS-DA) using Simca 14.1 software. The self-contained Winmuster software was used to gather and process the E-nose data, and Origin 2021 software was used to plot the radar map. Finally, a partial least squares regression (PLSR) model was developed using The Unscrambler X to determine the association between volatile compounds and E-nose sensors.

## 4. Conclusions

This study revealed that an increase in milk fat content has a positive impact on the physicochemical and textural properties of fermented milk, leading to significant improvements in water-holding capacity, dehydration shrinkage, color, hardness, springiness, gumminess, and chewiness, while showing minimal changes in pH and TTA. We employed the response surface methodology (RSM) to optimize the fermentation conditions and assess the interaction effects of independent factors (starter culture inoculation, milk fat content, fermentation time, and temperature) on the sensory evaluation of fermented milk. In this study, the optimal culture conditions were determined to be 25% milk fat content, 2.5% starter culture inoculation, 16 h of fermentation time, and a fermentation temperature of 30 °C. Additionally, using GC-IMS technology, we detected a total of 36 volatile compounds in fermented milk, with an increase in milk fat content leading to elevated levels of ketone compounds in the fermented milk. Through the calculation of the ROAV value, 14 volatile compounds including benzaldehyde, 2-heptanone-M, 2-heptanone-D, 3-hydroxy-2-butanone-M, 3-hydroxy-2-butanone-D, 2-pentanone-M, 2-pentanone-D, butanal, 2-propanone, propanal, 3-methylbutanal, 2-hexanone, nonanal, and octanal were identified as key aroma components (ROAV > 1). The electronic nose was able to significantly differentiate samples with different milk fat contents. These results and analyses provide valuable insights for the standardization and development of high-fat fermented milk products.

## Figures and Tables

**Table 1:** Texture profile analysis parameters for fermented milk with different concentrations of milk fat.

Milk Fat Content	Hardness/1/N	Cohesiveness	Springiness/mm	Gumminess/N	Chewiness/mj
5%	0.034 ± 0.006 ^d^	0.644 ± 0.032 ^ab^	9.564 ± 0.030 ^d^	0.023 ± 0.003 ^d^	0.264 ± 0.096 ^c^
10%	0.049 ± 0.004 ^c^	0.621 ± 0.014 ^b^	11.249 ± 0.200 ^c^	0.030 ± 0.002 ^c^	0.371 ± 0.063 ^c^
15%	0.060 ± 0.016 ^bc^	0.636 ± 0.007 ^ab^	13.794 ± 0.255 ^b^	0.043 ± 0.003 ^b^	0.645 ± 0.009 ^b^
20%	0.062 ± 0.006 ^bc^	0.664 ± 0.04 ^a^	14.067 ± 1.680 ^ab^	0.045 ± 0.004 ^b^	0.689 ± 0.069 ^b^
25%	0.070 ± 0.004 ^b^	0.650 ± 0.098 ^a^	14.995 ± 0.230 ^ab^	0.045 ± 0.003 ^b^	0.716 ± 0.212 ^b^
30%	0.103 ± 0.023 ^a^	0.664 ± 0.005 ^a^	15.432 ± 0.608 ^a^	0.065 ± 0.004 ^a^	1.033 ± 0.047 ^a^

^a–d^ Mean values with different superscript letters within a column differ significantly (*p* < 0.05).

**Table 2:** ANOVA analysis for the fitted quadratic polynomial model.

Source	Sum of Squares	df	Mean Square	F Value	*p* Value	Significance
Model	491.74	14	35.12	19.71	<0.0001	***
A—Content of cultures	10.08	1	10.08	5.66	0.0322	*
B—Content of fats	12.00	1	12.00	6.73	0.0212	*
C—Fermentation time	4.08	1	4.08	2.29	0.1524	
D—Fermentation temperature	33.33	1	33.33	18.70	0.0007	***
AB	4.00	1	4.00	2.24	0.1563	
AC	2.25	1	2.25	1.26	0.2801	
AD	0.0000	1	0.0000	0.0000	1.0000	
BC	0.2500	1	0.2500	0.1403	0.7136	
BD	30.25	1	30.25	16.97	0.0010	***
CD	2.25	1	2.25	1.26	0.2801	
A^2^	2.96	1	2.96	1.66	0.2187	
B^2^	182.21	1	182.21	102.24	<0.0001	***
C^2^	173.71	1	173.71	97.47	<0.0001	***
D^2^	165.42	1	165.42	92.82	<0.0001	***
Residual	24.95	14	1.78			
Lack of Fit	21.75	10	2.17	2.72	0.1737	
Pure Error	3.20	4	0.8000			
Cor Total	516.69	28				
R^2^	0.9517		Std. Dev.	1.33		
Adjusted R^2^	0.9034		Mean	78.90		
Predicted R^2^	0.7479		C.V. %	1.69		
Adeq Precision	16.4218					

Levels of significance: *, significant (*p* ≤ 0.05); ***, highly significant (*p* ≤ 0.001).

**Table 3:** Volatile compounds identified in different fermented milks by GC-IMS.

Count	Compound	CAS#	Formula	MW	RI ^a^	Rt [sec] ^b^	Dt ^c^ [RIPrel]	Comment
V1	2-Nonanone	C821556	C_9_H_18_O	142.2	1091.6	481.454	1.40809	
V2	3-Octanone	C106683	C_8_H_16_O	128.2	985.5	335.61	1.30991	
V3	Benzaldehyde	C100527	C_7_H_6_O	106.1	954.8	309.657	1.14748	
V4	2-Heptanone	C110430	C_7_H_14_O	114.2	888.3	254.655	1.26113	monomer
V5	2-Heptanone	C110430	C_7_H_14_O	114.2	888.3	254.655	1.63297	dimer
V6	Ethyl lactate	C97643	C_5_H_10_O_3_	118.1	832.7	225.752	1.15009	
V7	Cyclopentanone	C120923	C_5_H_8_O	84.1	792.5	204.866	1.1021	
V8	2,3-Butanediol	C513859	C_4_H_10_O_2_	90.1	790.5	203.795	1.38336	
V9	Methylpyrazine	C109080	C_5_H_6_N_2_	94.1	791.5	204.331	1.07755	
V10	3-Hydroxy-2-butanone	C513860	C_4_H_8_O_2_	88.1	703.4	167.648	1.06192	monomer
V11	3-Hydroxy-2-butanone	C513860	C_4_H_8_O_2_	88.1	703.4	167.648	1.32867	dimer
V12	2-Methylpropanoic acid	C79312	C_4_H_8_O_2_	88.1	766.5	193.353	1.1646	
V13	4-Methyl-3-penten-2-one	C141797	C_6_H_10_O	98.1	789.4	203.26	1.43582	
V14	2-Pentanone	C107879	C_5_H_10_O	86.1	664.8	155.331	1.11773	monomer
V15	2-Pentanone	C107879	C_5_H_10_O	86.1	665.8	155.598	1.36885	dimer
V16	2-Butanone	C78933	C_4_H_8_O	72.1	556.3	126.68	1.24385	
V17	Butanal	C123728	C_4_H_8_O	72.1	597.8	137.658	1.28961	
V18	2-Propanone	C67641	C_3_H_6_O	58.1	475.2	105.259	1.11326	
V19	Ethanol	C64175	C_2_H_6_O	46.1	419.4	90.532	1.04741	
V20	Propanal	C123386	C_3_H_6_O	58.1	516.7	116.237	1.04295	
V21	Hydroxyacetone	C116096	C_3_H_6_O_2_	74.1	625.2	144.888	1.04183	
V22	3-Methyl-2-butenal	C107868	C_5_H_8_O	84.1	773.8	196.298	1.09094	
V23	Ethyl Acetate	C141786	C_4_H_8_O_2_	88.1	598.9	137.926	1.09429	
V24	Pentanal	C110623	C_5_H_10_O	86.1	687.1	161.221	1.41684	
V25	Hexanal	C66251	C_6_H_12_O	100.2	784.8	200.85	1.25612	
V26	Heptanal	C111717	C_7_H_14_O	114.2	898.4	261.9	1.33202	
V27	(*E*)-2-Hexenal	C6728263	C_6_H_10_O	98.1	814.7	216.38	1.17688	
V28	Dimethyl sulfide	C75183	C_2_H_6_S	62.1	510.5	114.595	0.95577	
V29	2-Propanol	C67630	C_3_H_8_O	60.1	527.5	119.089	1.20946	
V30	1-Pentanol	C71410	C_5_H_12_O	88.1	756.8	189.41	1.25209	
V31	Isobutanol	C78831	C_4_H_10_O	74.1	601.6	138.652	1.16475	
V32	3-Methylbutanal	C590863	C_5_H_10_O	86.1	644.6	150.02	1.16267	
V33	1-Hexanol	C111273	C_6_H_14_O	102.2	868.3	244.257	1.32146	
V34	2-Hexanone	C591786	C_6_H_12_O	100.2	777.4	197.77	1.18483	
35	Nonanal	C124196	C_9_H_18_O	142.2	1109.1	506.666	1.48711	
36	Octanal	C124130	C_8_H_16_O	128.2	1004.2	355.979	1.41163	
37	1	unidentified	nd ^d^	nd	767.2	193.62	1.33537	
38	2	unidentified	nd	nd	775.7	197.10	1.41238	
39	3	unidentified	nd	nd	754.7	188.53	1.4001	
40	4	unidentified	nd	nd	721.8	175.15	1.40233	

^a^, retention time; ^b^, retention index; ^c^, drift time; ^d^, not detected in sample.

## Data Availability

The data presented in this study are available upon request from the corresponding author.

## Supplementary Materials

The following supporting information can be downloaded at https://www.mdpi.com/article/10.3390/molecules29051099/s1: Figure S1: Clustering heat map of the volatile components for the fermented milk samples; Figure S2: Flow chart for the manufacturing of fermented milk treatments; Table S1: Color changes of fermented milk with different concentrations of milk fat during storage; Table S2: The parameters and results of the RSM test; Table S3: The ROAV values of the main volatile compounds by GC-IMS; Table S4: Factors and levels of the RSM test; Table S5: Information on ten sensors of electronic nose sensors.
