# Supplementary material for: Exploring the Effect of Milk Fat on Fermented Milk Flavor Based on Gas Chromatography–Ion Mobility Spectrometry (GC-IMS) and Multivariate Statistical Analysis
Source: Molecules. 2024 Feb 29;29(5):1099. doi: 10.3390/molecules29051099 (PMC10934650; doi:10.3390/molecules29051099)
Supplement: Supplementary file 1 [file molecules-29-01099-s001.zip › molecules-2854788-supplementary.pdf]

Supplementary Materials for

Exploring the Effect of Milk Fat on Fermented Milk Flavor Based on Gas Chromatography–Ion Mobility Spectrometry (GC-IMS) and Multivariate Statistical Analysis

Table of Contents

Figure S1: The clustering heatmap of the volatile components for the fermented milk samples

Figure S2: Flow chart for the manufacturing of fermented milk treatments

Table S1: Color changes of fermented milk with different concentrations of milk fat during storage

Table S2: The parameters and results of RSM test

Table S3: The ROAV values of the main volatile compounds by GC-IMS

Table S4: Factors and levels of RSM test

Table S5: Information on ten sensors of electronic nose sensors.

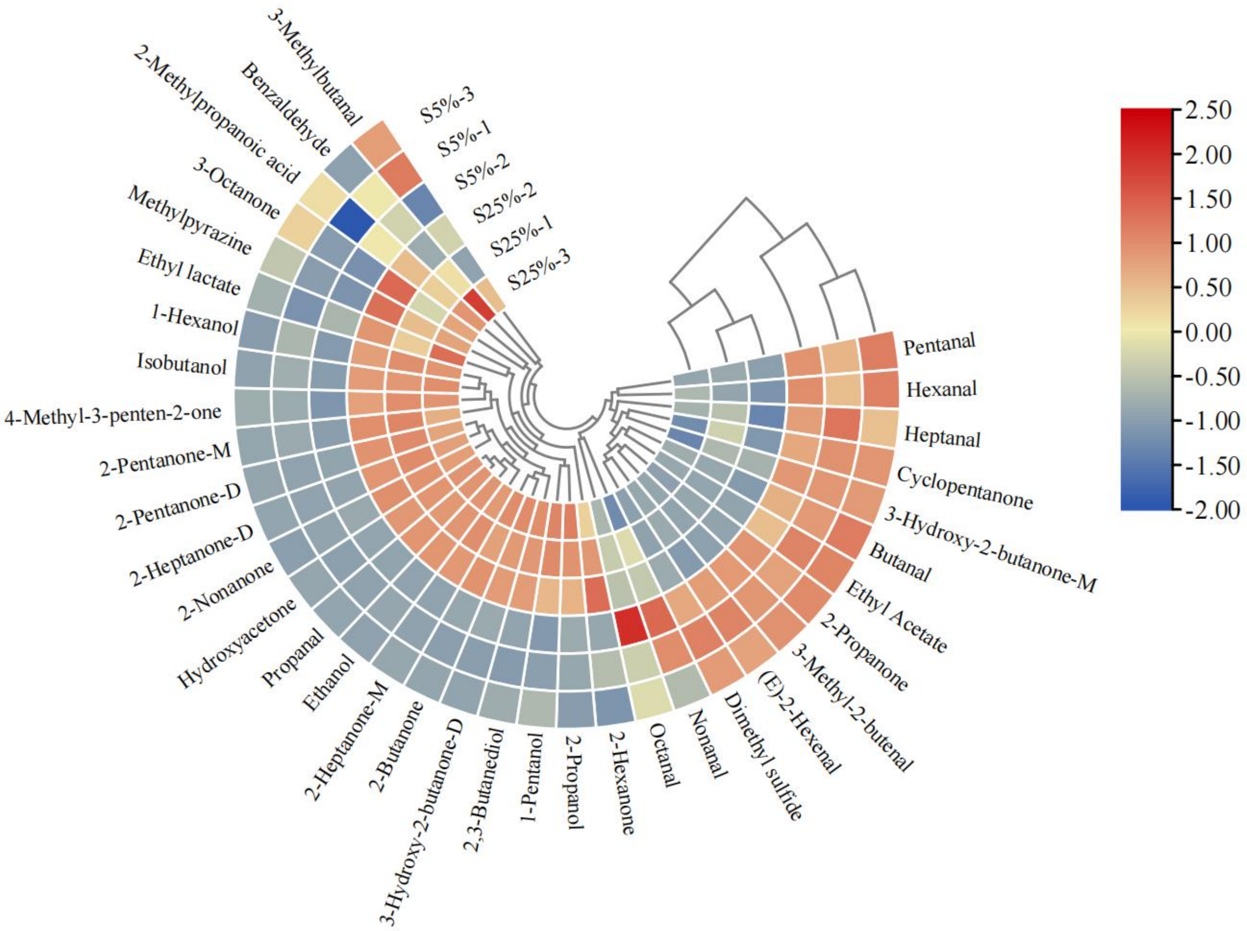

Figure S1: The clustering heatmap of the volatile components for the fermented milk samples.

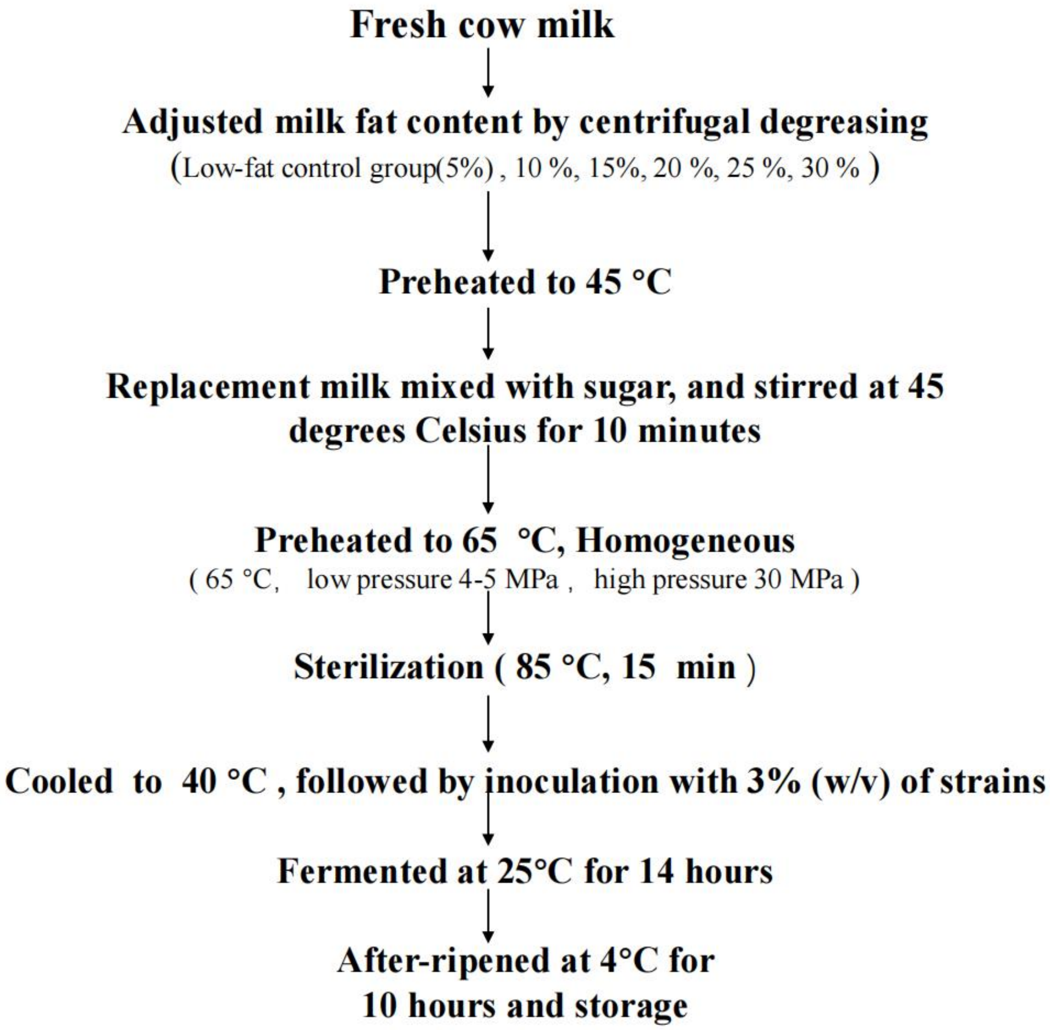

Figure S2: Flow chart for the manufacturing of fermented milk treatments.

Table S1. Influence of the milk fats content on color parameters difference parameter of fermented milks.

**Color**	**Days**	Milk fat content					
		5%	10%	15%	20%	25%	30%
	1	28.64±0.104^{d}	29.93±0.136^{c}	30.71±0.162^{b}	31.47±0.056^{a}	31.78±0.006	32.21±0.145
L^*	10	29.41±0.095^{d}	30.40±0.125^{c}	31.22±0.150^{b}	32.48±0.122	32.04±0.185^{a}	32.23±0.190^{a}
	20	29.67±0.068^{c}	31.09±0.029^{b}	31.23±0.017^{b}	32.99±0.528^{a}	32.33±0.081^{a}	32.33±0.139^{a}
	1	0.137±0.001^{a}	0.135±0.003^{a}	0.096±0.003^{b}	0.095±0.010^{b}	0.089±0.005^{b}	0.085±0.001^{b}
a^*	10	0.132±0.023^{a}	0.112±0.022^{a}	0.128±0.022^{a}	0.114±0.011^{a}	0.115±0.013^{a}	0.129±0.008^{a}
	20	0.055±0.009^{b}	0.097±0.004^{a}	0.031±0.013^{c}	0.034±0.006^{c}	0.032±0.004^{c}	0.022±0.001^{c}
	1	0.222±0.001^{b}	0.208±0.020^{b}	0.199±0.020^{bc}	0.174±0.014^{d}	0.1817±0.005^{cd}	0.261±0.003^{a}
b^*	10	0.229±0.019^{b}	0.232±0.009^{c}	0.235±0.025^{bc}	0.248±0.024^{bc}	0.271±0.024^{ab}	0.294±0.021^{a}
	20	0.193±0.022^{b}	0.154±0.007^{c}	0.174±0.020^{bc}	0.182±0.016^{bc}	0.208±0.007^{ab}	0.229±0.031^{a}

Values are mean ± SD (n = 3), a, b, c, d—values followed by the same letter within a column do not differ significantly according to Tukey's test ($p < 0.05$).

Table S2. Box-Behnken design matrix with experimental runs and observed responses.

Runs	A: Content of cultures/%	B:Content of fats/%	C:Fermentation time/h	D:Fermentation temperature/°C	Sensory Score
1	0	0	0	0	85
2	-1	0	1	0	82
3	0	1	1	0	76
4	0	-1	0	-1	70
5	-1	0	0	1	82
6	-1	1	0	0	82
7	0	1	-1	0	74
8	0	0	0	0	87
9	0	0	1	-1	74
10	-1	0	-1	0	82
11	0	-1	0	1	80
12	0	0	-1	-1	75
13	0	1	0	-1	78
14	0	-1	1	0	74
15	-1	0	0	-1	78
16	0	0	1	1	77
17	1	-1	0	0	79
18	-1	-1	0	0	78
19	1	1	0	0	79
20	0	0	0	0	85

21	1	0	1	0	81
22	0	1	0	1	77
23	0	0	0	0	86
24	0	0	-1	1	75
25	0	-1	-1	0	73
26	1	0	-1	0	78
27	1	0	0	1	80
28	0	0	0	0	85
29	1	0	0	-1	76

Table S3. Relative odor activity values (ROAV) and odor characteristics of volatile compounds.

Compound	Threshold value (ug/kg) [a]	Relative content（%）		ROAV		Odor[b]
		S5%	S25%	S5%	S25%	
2-Nonanone	5-200	0.337	0.557	0.34	0.51	butter, fruity
3-Octanone	18	0.207	0.187	0.058	0.048	butter
Benzaldehyde	0.5	0.160	0.130	1.584	1.185	almondy
2-Heptanone-M	9	2.693	3.553	1.48	1.798	banana，peach
2-Heptanone-D	9	1.400	4.413	0.77	2.250	banana，peach
Ethyl lactate	14000	0.060	0.080	＜ 0.001	＜ 0.001	fruity
Cyclopentanone	840-1260	1.600	1.130	0.009	0.006	mint
2,3-Butanediol	1000	0.673	0.930	0.003	0.004	sweet
Methylpyrazine	6	0.100	0.090	0.083	0.068	roast, nut
3-Hydroxy-2-butanone-M	0.75	9.723	6.933	64.152	42.134	butter
3-Hydroxy-2-butanone-D	0.75	15.163	16.440	100	100	butter
2-Methylpropanoic acid	10	0.353	0.510	0.173	0.233	fatty, butyric acid
4-Methyl-3-penten-2-one	nd	0.313	0.380	nd	nd	penetrating odor
2-Pentanone-M	2.3	1.500	1.357	3.228	2.696	sweet, banana
2-Pentanone-D	2.3	1.877	4.410	4.046	8.743	sweet, banana
2-Butanone	50000	19.380	21.333	0.002	0.002	fruity, sweet
Butanal	0.07	1.237	0.637	87.69	41.691	stimulating
2-Propanone	50-100	34.573	16.940	1.71	0.77	fruity, sweet
Ethanol	5000-	1.780	9.137	0.002	0.008	spicy, wine

	13000					
Propanal	9.5-37	0.600	3.723	0.313	1.786	solvent
Hydroxyacetone	nd	1.380	3.997	nd	nd	nd
3-Methyl-2-butenal	nd	0.523	0.080	nd	nd	nd
Ethyl Acetate	1-50	0.317	0.160	0.211	0.097	pineapple
Pentanal	9	0.447	0.107	0.248	0.056	nut, almond
Hexanal	4.5	0.330	0.173	0.363	0.172	grassy
Heptanal	3	0.213	0.143	0.347	0.213	fruity, grassy
(E)-2-Hexenal	17	0.307	0.127	0.09	0.035	fruity
Dimethyl sulfide	nd	0.563	0.217	nd	nd	cooked flavor
2-Propanol	200-500	0.260	0.307	0.006	0.007	woody
1-Pentanol	20	0.153	0.230	0.037	0.052	spicy, wine
Isobutanol	500	0.207	0.400	0.002	0.004	oil, alcohol
3-Methylbutanal	0.2-2	0.167	0.123	2.104	1.368	apple, peach
1-Hexanol	250	0.120	0.197	0.002	0.004	floral, resin
2-Hexanone	0.09	0.117	0.137	6.6	7.918	acetone
Nonanal	1	0.957	0.643	4.752	2.918	flowery, fatty
Octanal	0.7	0.210	0.090	1.485	0.586	oil, citrus

a, Threshold value referenced the ' Compilations of flavor threshold values in water and other media ' and the literature reference; b, Odor descriptions were cited from recent reports.

Table S4. Factors and levels of RSM test.

Levels	A:Content of cultures(%)	B:Content of fats(%)	C:Fermentation time(h)	D:Fermentation temperature(℃)
-1	2	20	14	25
0	3	25	16	30
1	4	30	18	35

Table S5. E-nose sensors and their main application in PEN3.

Sensor name	Performance description	Representative material species
W1C	Sensitive to aromatic constituents, benzene	Aromatic
W5S	Sensitive to nitrogen oxides	Broad range
W3C	Sensitive to aroma, ammonia	Aromatic compounds
W6S	Mainly selective for hydrides	Hydrogen
W5C	Short-chain alkane aromatic components	Arom-aliph
W1S	Sensitive to methyl	Broad-methane
W1W	Sensitive to sulfides	Sulphur-organic

W2S	Sensitive to alcohols, aldehydes and ketones	Broad-alcohol
W2W	Sensitive to aromatic ingredients and organic sulfides	Sulph-chlor
W3S	Sensitive to long-chain alkanes	Methane-aliph
